# Is early vaginal sexual behavior associated with the occurrence of pelvic inflammatory disease: An analysis based on NHANES

**DOI:** 10.1097/MD.0000000000048494

**Published:** 2026-04-24

**Authors:** Qilin Cheng, Guoxia Mao, Aiying Ding, Suqin Liu, Le Bo, Xin Qi, Tingting Bi, Buze Chen, Wei Jin

**Affiliations:** a Gynecology, The Affiliated Hospital of XuZhou Medical University, XuZhou, Jiangsu, China; b Gynecology, Huai’an Hospital Affiliated to Yangzhou University (The Fifth People’s Hospital of Huai’an), Huai’an, Jiangsu, China.

**Keywords:** NHANES data, PID, premature sexual activity

## Abstract

Pelvic inflammatory disease (PID), also referred to as pelvic inflammatory disorder, is a group of inflammatory conditions affecting the upper female reproductive tract and its surrounding tissues, including the uterus, fallopian tubes, ovaries, and pelvic peritoneum. The incidence of PID is ubiquitous among young sexually active women, and is rare among women who are not sexually active. However, there is a lack of relevant cross-sectional studies about this. Data in this study were collected and screened from the National Health And Nutrition Examination Surveys from 2013 to 2016. The variables were extracted from interviews and compared between the age of first sexual experience and the PID. The data was analyzed by weighted multivariate logistic regression. After excluding individuals who were not eligible and had invalid data, we finally identified 3437 participants for inclusion in this study. We found a positive association between the age of first sexual experience and the PID (*P* < .05). In conclusion, a positive correlation between the age of first sexual experience and PID was found. The results can help us developing more effective strategies to prevent and manage both of these important health issues. Our findings provide a better understanding to improve young female adolescents’ correct understanding of premature sexual behavior and advise them against engaging in it prematurely.

## 1. Introduction

Pelvic inflammatory disease (PID), also referred to as pelvic inflammatory disorder, is a group of inflammatory conditions affecting the upper female reproductive tract and its surrounding tissues, including the uterus, fallopian tubes, ovaries, and pelvic peritoneum.^[1]^ PID occurs when the natural defense mechanisms of the body are impaired or the immune system is weakened, allowing certain bacteria to cause inflammation.The inflammation can be localized to 1 area or involve multiple areas simultaneously. It is often a sexually transmitted disease but other etiologic routes are also note.^[2]^

A variety of risk factors have been identified including adolescence, young adulthood, adolescent cervical, ectropion, multiple sexual partners, immature immune system, history of previous PID, risky contraceptive practices and others.^[3]^ The primary symptoms of PID include lower abdominal pain, fever, abnormal vaginal discharge, or abnormal vaginal bleeding.^[4]^ In the early stages, the symptoms may be subtle or absent, but they tend to worsen as the disease progresses. Nowadays, as the improving of living standards and people’s awareness of health, the incidence rate of PID is increasing year by year. And the cost for this is rising annually.^[5]^In the United States, approximately 0.5 to 1 million cases of PID develop annually, and the average cost is up to $3025 per episode for PID therapy.^[5,6]^ Meanwhile, the majority of PID patients are prone to experience disease recurrence. It is also added burdens on society and healthcare systems.^[7]^ Hence, it is crucial to examine the risk factors associated with PID to enable early intervention.

Previous studies^[1]^ have shown that, the incidence of PID is ubiquitous among young sexually active women, and is rare among women who are not sexually active. Although only about half of female adolescents are sexually active, they have the highest age-specific rates of PID among sexually experienced women. The risk of developing PID for a 15 years old sexually active girl is estimated to be ten times that of a 24 years old women.^[8]^ Premature sexual activity can have numerous negative consequences for female adolescents. These include an increased risk of unintended pregnancy, sexually transmitted infections (STIs), and long-term psychological distress. Young girls may not be fully aware of the risks associated with sexual activity, and they may lack the maturity and experience to make informed decisions about their sexual health.^[9,10]^ However, there is few research about the association between the age of first sexual experience and PID.

In this study, we collect the data from the web of the National Health and Nutrition Examination Surveys (NHANES), and want to find the association between the age of first sexual experience and PID, so as to provide a preventive measures for PID.

## 2. Materials and methods

**Figure 1.**
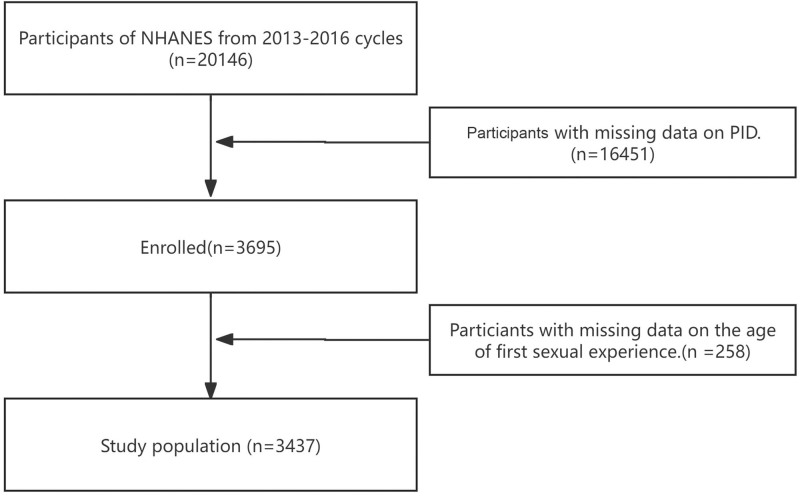
Participant selection flowchart for the study using NHANES 2013 to 2016 data. The diagram outlines the inclusion and exclusion process, beginning with 20,146 initially screened individuals. Following the exclusion of participants with missing or invalid data on age at first sexual intercourse and PID status, the final analytical cohort consisted of 3437 sexually active individuals. n = number of participants, NHANES = National Health and Nutrition Examination Surveys, PID = pelvic inflammatory disease.

NHANES is a survey conducted by the Centers for Disease Prevention and Control in the United States (CDC). The CDC organizes a large-scale health survey project every 2 years, and its data is publicly available worldwide for free use. Use of the dataset from the NHANES was approved by the National Center for Health. The study was conducted in accordance with the revised Declaration of Helsinki. All informed consents were obtained prior to data collection. We searched and downloaded the health data of the 2013 to 2016 cycles on its website. The recruitment details, recruitment procedures, population characteristics, and research design of the surveyed population were provided by the CDC. All participants completed a family interview, and the observers came from a unified team and implemented a unified interview standard, which significantly reduced the differences caused by observers (Fig. 1).

### 2.1. Study variables

The NHANES data were collected through standardized questionnaires and medical data. Combining the characteristics of previous epidemiological studies of PID and the variables provided by the NHANES data, we collected the following data from the subjects as variables: age, race, education level, marital status, PID, average cigarettes, average alcoholic drinks, diabetes, body mass index (BMI), monthly family income, age of first sexual experience. All details of study variables in this study could be accessed in www.cdc.gov/nchs/nhanes.

### 2.2. Statistical analysis

After data screening, continuous variables were expressed as mean standard, enumeration data were expressed as percentage (%), *t* test was used for continuous variables, and chi-square test was used for categorical variables. The data analysis in this study took into account sampling weights based on the analytical guideline edited by the National Center for Heath Statistics, and was conducted using package R version 3.43 (The R Foundation for Statistical Computing, Vienna, Austria, http://www.R-project.org) and EmpowerStats software (X&Y Solutions Inc., Boston, http://www.empowerstats.com). The significance level was 0.05.

## 3. Results

The data showed that a total of 20,146 people were recruited to participate in the questionnaire, physical examination and evaluation. After excluding invalid values with the age of first sexual experience and PID, a total of 3437 people were included in this study. The relevant information of the above-mentioned people was used for further research. The datasets generated and analyzed during the current study are available in the NHANES data (www.cdc.gov/nchs/nhanes), or required from the corresponding author.

Among 3437 sexually active people who were included in this study, as shown in the Table 1. We categorized the continuous variable age of first sexual experience into 4 groups. There was a significant difference among the age, race, BMI, education level, average alcoholic drinks, monthly family income and the age of first sexual experience (*P* < .05).

**Table 1:** Characteristic distribution of the participants in NHANES 2013 to 2016 across the the age of sexual experience.

	Age of first sexual experience				*P* value
	Q1	Q2	Q3	Q4	
Age	37.37 ± 11.74	38.07 ± 12.33	39.64 ± 12.19	41.07 ± 11.17	< .0001
Race					< .0001
Mexican American	11.36	8.81	9.25	12.12	
Other Hispanic	5.68	5.03	7.03	8.41	
Non-Hispanic White	54.7	67.92	63.04	57.95	
Non-Hispanic Black	20.9	12.87	13.87	7.92	
Other Race - Including MultiRacial	7.35	5.36	6.8	13.59	
Marital status					< .0001
Married	39.57	47.58	53	67.47	
Widowed	2.23	2.03	2.3	1.58	
Divorced	12.66	12.08	12.15	8.71	
Separated	5.93	3.82	2.55	2.17	
Never married	23.62	20.97	20.66	15.69	
Living with partner	15.99	13.52	9.34	4.37	
Average cigarettes	10.57 ± 8.04	11.31 ± 7.62	17.47 ± 77.49	8.69 ± 5.97	.2998
BMI	30.92 ± 8.73	29.16 ± 7.53	30.11 ± 7.89	28.86 ± 8.17	< .0001
Education level					< .0001
< 9th grade	3.98	2.5	2.68	4.22	
9–11th grade	13.03	10.01	6.38	5.74	
High school graduate	19.26	23.35	17.21	12.2	
Some college or AA degree	47.25	38.2	38.88	28.48	
College graduate or above	16.47	25.93	34.85	49.36	
PID					< .0001
Yes	10	7.15	3.79	1.68	
No	90	92.85	96.21	98.32	
Average alcoholic drinks					< .0001
1	23.86	28.19	35.78	52.42	
2	31.52	37.36	37.26	30.43	
3	22.29	15.95	13	11.28	
4	7.51	8	7.17	3.14	
5	5.82	5.64	3.11	1.72	
6	3.22	2.56	1.25	0.84	
7	1.4	0.16	0.32	0.1	
8	1.66	1.01	1.15	0.06	
9	0.3	0.73	0.26		
10	1.65	0.18	0.06	52.42	
11	0.22	0.14	0.3	30.43	
12	0.18	0.09	0.17	11.28	
13	0.22		0.16	3.14	
15	0.17	28.19		1.72	
16		37.36	35.78	0.84	
18	23.86	15.95	37.26	0.1	
20	31.52	8	13	0.06	
Diabetes					.6727
Yes	6.79	5.45	6.53	6.96	
No	91.34	92.69	91.84	90.39	
Borderline	1.78	1.79	1.54	2.65	
Don’t know	0.1	0.07	0.1		
Monthly family income					< .0001
1	3.87	4.32	1.96	2.86	
2	5.87	4.62	3.31	2.05	
3	9.54	7.03	7.24	4.35	
4	8.45	5.52	5.54	3.81	
5	9.38	6.11	6.72	4.74	
6	9.11	8.13	6.74	6.22	
7	12.81	9.99	10.02	10.59	
8	7.69	8.17	9.49	9.52	
9	4.97	6.39	6.84	7.95	
10	2.92	5.74	7.23	6.24	
11	6.27	8.39	8.05	11.47	
12	15.14	18.55	19.44	22.38	
77	0.5	1.82	1.6	2.7	
99	3.47	5.23	5.8	5.14	

BMI = body mass index, NHANES = National Health and Nutrition Examination Surveys, PID = pelvic inflammatory disease.

In Table 2, the impact of the age of sexual experience and the PID was presented. It is significant association between them (*P* < .001).The age of sexual experience was associated with the PID in the crude model, and the odds ratio (OR) for the PID across the quartiles of the age of sexual experience were 0, 0.0 (95% confidence interval [CI]: 0.0, 0.1), 0.1 (95% CI: 0.0, 0.1), 0.1 (95% CI: 0.1, 0.1), the *P* for trend is < .001. Next, we examined the association between the age of first sexual experience and PID, taking into account both age and race. As counted with age, the *P* for trend is < .001. After adjusted race, the OR were 0, 0.0 (95% CI: 0.0, 0.1), 0.1 (95% CI: 0.0, 0.1), 0.1 (95% CI: 0.1, 0.1). The *P* for trend is < .001. Furthermore, we adjusted for the age, race, education level, diabetes, marital status, BMI, average cigarettes, average alcoholic drinks and monthly family income, the OR were 0, 0.0 (95% CI: −0.1, 0.1), 0.1 (95% CI: −0.0, 0.2), 0.1 (95% CI: 0.0, 0.3). The *P* for trend was significant (*P* = .007).

**Table 2:** Association between the age of sexual experience and PID among US in NHANES 2013 to 2016.

Exposure	Crude model	Model 1	Model 2
Age of sexual experience			
Q1	0	0	0
Q2	0.0 (0.0, 0.1) 0.020	0.0 (0.0, 0.1) 0.024	0.0 (−0.1, 0.1) 0.745
Q3	0.1 (0.0, 0.1) < 0.001	0.1 (0.0, 0.1) < 0.001	0.1 (−0.0, 0.2) 0.059
Q4	0.1 (0.1, 0.1) < 0.001	0.1 (0.1, 0.1) < 0.001	0.1 (0.0, 0.3) 0.023
*P* for trend	< .001	< .001	.007

Crude model adjusted for: none.

Model 1 adjusted for: age; race.

Model 2 model adjusted for: age; race; education level; marital status; average cigarettes; average alcoholic drinks; diabetes; body mass index; monthly family income.

NHANES = National Health and Nutrition Examination Surveys, PID = pelvic inflammatory disease, US = United States.

Upon conducting a grouped analysis of data based on age, it was found that in crude model, the OR of the total is 0, 0.0 (95% CI: 0.0, 0.1), 0.1 (95% CI: 0.0, 0.1), 0.1 (95% CI: 0.1, 0.1), the *P* for trend is < .001. In model 2, the OR is 0, 0.0 (95% CI: 0.0, 0.1), 0.1 (95% CI: 0.0, 0.1), 0.1 (95% CI: 0.1, 0.1), the *P* for trend is < .001. When further adjusted, the OR is 0, 0.0 (95% CI: −0.1, 0.1), 0.1 (95% CI: 0.0, 0.2), 0.2 (95% CI: 0.0, 0.3), the *P* for trend is .005. After conducted a grouped analysis of data based on race, we find the association among the Non-Hispanic Black is significant. The OR is 0, 0.0 (95% CI: −0.0, 0.1), 0.1 (95% CI: 0.0, 0.1), 0.1 (95% CI: 0.0, 0.1), the *P* for trend is < .001. When adjusted the age, the OR come to 0, 0.1 (95% CI: −0.0, 0.1), 0.1 (95% CI: 0.0, 0.2), 0.1 (95% CI: 0.0, 0.2), the *P* for trend is < .001. When further adjusted, the OR come to 0, 0.1 (95% CI: −0.1, 0.3), 0.3 (95% CI: 0.1, 0.5), 0.3 (95% CI: −0.0, 0.6), the *P* for trend is .001. (Table 3)

**Table 3:** Subgroup analysis of association between the age of sexual experience and PID among US in NHANES 2013 to 2016.

	Crude model					Model 1					Model 2				
	Age of first sexual experience				*P* for trend	Age of first sexual experience				*P* for trend	Age of first sexual experience				*P* for trend
	Q1	Q2	Q3	Q4		Q1	Q2	Q3	Q4		Q1	Q2	Q3	Q4	
Age Total	0	0.0 (0.0, 0.1) 0.016	0.1 (0.0, 0.1) < 0.001	0.1 (0.1, 0.1) < 0.001	< .001	0	0.0 (0.0, 0.1) 0.025	0.1 (0.0, 0.1) < 0.001	0.1 (0.1, 0.1) < 0.001	< .001	0	0.0 (−0.1, 0.1) 0.613	0.1 (0.0, 0.2) 0.043	0.2 (0.0, 0.3) 0.018	.005
Age quartile = Q1	0	0.0 (0.0, 0.1) 0.014	0.1 (0.0, 0.1) < 0.001	0.1 (0.0, 0.1) < 0.001	.026	0	0.0 (0.0, 0.1) 0.018	0.1 (0.0, 0.1) < 0.001	0.1 (0.0, 0.1) < 0.001	.044	0	0.1 (−0.1, 0.3) 0.301	0.2 (−0.0, 0.4) 0.098	0.1 (−0.2, 0.5) 0.531	.15
Age quartile = Q2	0	−0.0 (−0.1, 0.0) 0.680	0.0 (−0.0, 0.1) 0.412	0.0 (−0.0, 0.1) 0.119	< .001	0	−0.0 (−0.1, 0.0) 0.582	0.0 (−0.0, 0.1) 0.477	0.0 (−0.0, 0.1) 0.180	< .001	0	−0.1 (−0.2, 0.0) 0.165	−0.1 (−0.2, 0.1) 0.361	0.0 (−0.2, 0.2) 0.947	.487
Age quartile = Q3	0	0.1 (0.0, 0.2) < 0.001	0.1 (0.0, 0.1) < 0.001	0.1 (0.1, 0.2) < 0.001	< .001	0	0.1 (0.1, 0.2) < 0.001	0.1 (0.0, 0.1) < 0.001	0.1 (0.1, 0.2) < 0.001	< .001	0	0.1 (−0.1, 0.3) 0.342	0.0 (−0.2, 0.2) 0.858	0.1 (−0.2, 0.4) 0.480	.59
Age quartile = Q4	0	0.0 (−0.1, 0.1) 0.900	0.1 (0.0, 0.1) 0.009	0.1 (0.1, 0.2) < 0.001	< .001	0	0.0 (−0.1, 0.1) 0.950	0.1 (0.0, 0.1) 0.011	0.1 (0.1, 0.2) < 0.001	< .001	0	0.0 (−0.2, 0.2) 0.858	0.2 (0.0, 0.4) 0.040	0.1 (−0.2, 0.4) 0.434	.008
Race Total	0	0.0 (0.0, 0.1) 0.028	0.1 (0.0, 0.1) < 0.001	0.1 (0.1, 0.1) < 0.001	< .001	0	0.0 (0.0, 0.1) 0.024	0.1 (0.0, 0.1) < 0.001	0.1 (0.1, 0.1) < 0.001	< .001	0	0.0 (−0.1, 0.1) 0.745	0.1 (−0.0, 0.2) 0.059	0.1 (0.0, 0.3) 0.023	.007
Race = Mexican American	0	0.0 (−0.0, 0.1) 0.057	0.0 (−0.0, 0.1) 0.077	0.0 (0.0, 0.1) 0.040	.109	0	0.0 (−0.0, 0.1) 0.059	0.0 (−0.0, 0.1) 0.073	0.0 (0.0, 0.1) 0.031	.076	0	0.2 (,) NaN	0.4 (,) NaN	1.4 (,) NaN	NaN
Race = Other Hispanic	0	−0.0 (−0.1, 0.0) 0.226	−0.0 (−0.1, 0.1) 0.785	0.0 (−0.1, 0.1) 0.875	.29	0	−0.0 (−0.1, 0.0) 0.281	−0.0 (−0.1, 0.1) 0.892	0.0 (−0.1, 0.1) 0.692	.197	0	−0.5 (−1.1, 0.0) 0.306	−0.0 (−0.4, 0.3) 0.894	0.2 (−0.4, 0.8) 0.685	.907
Race = Non-Hispanic White	0	0.0 (−0.0, 0.1) 0.567	0.0 (0.0, 0.1) 0.028	0.1 (0.0, 0.1) < 0.001	< .001	0	0.0 (−0.0, 0.1) 0.564	0.0 (0.0, 0.1) 0.020	0.1 (0.0, 0.1) < 0.001	< .001	0	−0.0 (−0.1, 0.1) 0.744	0.0 (−0.1, 0.1) 0.473	0.1 (−0.1, 0.3) 0.197	.145
Race = Non-Hispanic Black	0	0.0 (−0.0, 0.1) 0.064	0.1 (0.0, 0.1) < 0.001	0.1 (0.0, 0.1) 0.005	< .001	0	0.1 (−0.0, 0.1) 0.062	0.1 (0.0, 0.2) < 0.001	0.1 (0.0, 0.2) 0.003	< .001	0	0.1 (−0.1, 0.3) 0.235	0.3 (0.1, 0.5) < 0.001	0.3 (−0.0, 0.6) 0.089	.001
Race = Other Race - Including Multiracial	0	0.1 (0.1, 0.2) < 0.001	0.2 (0.1, 0.3) < 0.001	0.2 (0.1, 0.2) < 0.001	< .001	0	0.1 (0.1, 0.2) < 0.001	0.2 (0.1, 0.3) < 0.001	0.2 (0.1, 0.3) < 0.001	< .001	0	0.3 (−0.1, 0.7) 0.151	−0.0 (−0.6, 0.5) 0.987	0.5 (−0.9, 1.9) 0.494	.613

Crude model adjusted for: none.

Model 1 adjusted for: age; race.

Model 2 model adjusted for: age; race; education level; marital status; average cigarettes; average alcoholic drinks; diabetes; body mass index; monthly family income.

NHANES = National Health and Nutrition Examination Surveys, PID = pelvic inflammatory disease, US = United States.

## 4. Discussion

The present study, utilizing data from the NHANES, explores the correlation between early vaginal sexual behavior and the occurrence of PID. The findings indicate a significant association between the age of first sexual experience and PID prevalence, highlighting a critical public health issue among young, sexually active women. Evidence supports that young age at first intercourse is a risk factor for PID.^[11]^ Additionally, PID can be caused by infections such as Mycoplasma genitalium, which are often acquired through sexual intercourse or cervical mucus barrier disruption.^[12]^ The study’s results align with these insights, underscoring the importance of considering sexual behavior in PID risk assessment and prevention strategies.

The biological plausibility of our results is supported by existing literature highlighting the vulnerability of the female reproductive system to infections during adolescence. The immature epithelial barrier of the cervix in younger women is more susceptible to ascending infections, potentially leading to PID.^[13]^ Furthermore, early sexual debut may coincide with a higher likelihood of engaging in unprotected sex and having multiple sexual partners, both of which are established risk factors for PID.^[14]^

Our study’s findings align with previous research indicating that sexually active adolescent females bear a disproportionately high burden of PID, despite comprising only a fraction of the sexually experienced population. The elevated risk of PID in this demographic may also be attributed to an underdeveloped immune system and a lack of awareness regarding sexual health and safe practices. This is supported by evidence showing that PID is most common among young women, particularly those younger than age 25 years, and those with risk factors such as infection with an STI (most often gonorrhea or chlamydia) and multiple sex partners.^[15]^ Additionally, the immature immune system and failure to consistently use condoms are key factors contributing to the development of PID in adolescents.^[2]^

It is important to acknowledge the limitations inherent in our study’s design. The cross-sectional nature of the NHANES data limits our ability to infer causality and establish a temporal sequence between early sexual activity and PID development. Longitudinal studies are necessary to provide a more definitive understanding of this relationship.^[16]^

Additionally, the reliance on self-reported data introduces the potential for recall bias, which may affect the accuracy of our findings. Despite these limitations, the large, nationally representative sample of NHANES data strengthens the generalizability of our results.

The implications of our findings are 2-fold. Firstly, they highlight the need for targeted sexual health education and interventions aimed at delaying the onset of sexual activity among adolescents. Secondly, they emphasize the importance of promoting safe sex practices to reduce the risk of PID and other STIs.

## 5. Conclusion

In conclusion, a positive correlation between the age of first sexual experience and PID was found. The results can help us develop more effective strategies to prevent and manage both of these important health issues. Our findings provide a better understanding to improve young female adolescents’ correct understanding of premature sexual behavior and advise them against engaging in it prematurely.

## Acknowledgments

We thank the editor and series editor for the constructive criticisms of an earlier version of this article.

## Author contributions

**Conceptualization:** Qilin Cheng, Wei Jin.

**Data curation:** Qilin Cheng, Le Bo, Xin Qi, Wei Jin.

**Formal analysis:** Qilin Cheng, Guoxia Mao, Suqin Liu, Xin Qi, Wei Jin.

**Funding acquisition:** Qilin Cheng.

**Investigation:** Qilin Cheng, Guoxia Mao, Aiying Ding, Suqin Liu, Wei Jin.

**Methodology:** Qilin Cheng, Guoxia Mao, Aiying Ding, Suqin Liu, Le Bo, Xin Qi, Tingting Bi, Wei Jin.

**Project administration:** Aiying Ding, Suqin Liu, Le Bo, Tingting Bi, Wei Jin.

**Resources:** Guoxia Mao, Aiying Ding, Suqin Liu, Le Bo, Xin Qi, Tingting Bi.

**Software:** Guoxia Mao, Suqin Liu, Le Bo, Xin Qi, Tingting Bi.

**Supervision:** Tingting Bi.

**Validation:** Xin Qi.

**Writing – original draft:** Guoxia Mao, Aiying Ding, Le Bo, Wei Jin.

**Writing – review & editing:** Buze Chen, Wei Jin.
